# Exposure to ozone impacted Th1/Th2 imbalance of CD^4+^ T cells and apoptosis of ASMCs underlying asthmatic progression by activating lncRNA PVT1-miR-15a-5p/miR-29c-3p signaling

**DOI:** 10.18632/aging.104124

**Published:** 2020-11-20

**Authors:** Yangyang Wei, Baofen Han, Wenjuan Dai, Shufang Guo, Caiping Zhang, Lixuan Zhao, Yan Gao, Yi Jiang, Xiaomei Kong

**Affiliations:** 1 Department of Respiratory and Critical Care Medicine, The First Hospital of Shanxi Medical University, Taiyuan 030001, China; 2 Department of Medicine, Shanxi Medical University, Taiyuan 030001, China

**Keywords:** asthma, ozone, lncRNA PVT1, miR-15a-5p, miR-29c-3p

## Abstract

This investigation attempted to elucidate whether lncRNA PVT1-led miRNA axes participated in aggravating ozone-triggered asthma progression. One hundred and sixty-eight BALB/c mice were evenly divided into saline+air group, ovalbumin+air group, saline+ozone group and ovalbumin+ozone group. Correlations were evaluated between PVT1 expression and airway smooth muscle function/inflammatory cytokine release among the mice models. Furthermore, pcDNA3.1-PVT1 and si-PVT1 were, respectively, transfected into CD^4+^T cells and airway smooth muscle cells (ASMCs), and activities of the cells were observed. Ultimately, a cohort of asthma patients was recruited to estimate the diagnostic performance of PVT1. It was demonstrated that mice of ovalbumin+ozone group were associated with higher PVT1 expression, thicker trachea/airway smooth muscle and smaller ratio of Th1/Th2-like cytokines than mice of ovalbumin+air group and saline+ozone group (*P*<0.05). Moreover, pcDNA3.1-PVT1 significantly brought down Th1/Th2 ratio in CD^4+^ T cells by depressing miR-15a-5p expression and activating PI3K-Akt-mTOR signaling (*P*<0.05). The PVT1 also facilitated ASMC proliferation by sponging miR-29c-3p and motivating PI3K-Akt-mTOR signaling (*P*<0.05). Additionally, PVT1 seemed promising in diagnosis of asthma, with favorable sensitivity (i.e. 0.844) and specificity (i.e. 0.978). Conclusively, lncRNA PVT1-miR-15a-5p/miR-29c-3p-PI3K-Akt-mTOR axis was implicated in ozone-induced asthma development by promoting ASMC proliferation and Th1/Th2 imbalance.

## INTRODUCTION

Asthma is clinically embodied as repetitive wheeze, dyspnea, chest stress and cough at early morning or in the night, and its global incidence is expected to reach 400 million by 2025 [1]. Even if glucocorticoid-based treatments were efficacious in relieving inflammation of asthma [2], their effects on patients with airflow limitation were not so desirable, which led to skyrocketing mortality [3]. It was widely acknowledged that airway smooth muscle cell (ASMC) and CD^4+^ T cell played crucial roles in airway inflammation and airway remodeling, which exacerbated airflow limitation [4–7]. Specifically, excessive proliferation of ASMCs engendered airway remodeling [8], and promoted airway inflammation by stimulating production of interleukins (e.g. IL-6), chemokines and cell adhesion factors [9]. Biased differentiation of CD^4+^ T cells into Th1-like and Th2-like cells was also responsible for abnormal inflammation in asthma [10]. Taken together, asthma treatment might be improved by restraining ASMC over-proliferation and by preventing Th1/Th2 imbalance.

LncRNAs, a group of ncRNAs with length of > 200 nucleotides, demonstrated huge potential in urging or blocking asthma progression by acting upon miRNAs that mattered in asthmatic inflammation or airway remodeling [11]. For example, knockout of miR-155 was reported to mitigate airway inflammation and airway hyper-responsiveness in ovalbumin (OVA)-sensitized mice [12], so it was probable that lncRNA MALAT1 elevated asthma risk by sponging miR-155 and inhibiting its expression [13]. Of note, expression of lncRNA PVT1 was dramatically lowered in ASMCs that were exposed to anti-asthmatic drugs [14], providing a hint that PVT1 might be associated with ASMC dysfunction underlying asthma etiology. Suppressing PVT1 expression also engendered a marked decrease of IL-6 level, which was reflective of abated inflammation in asthma [15]. Despite hidden linkages between PVT1 and airway remodeling/inflammation, it remained ambiguous whether PVT1 indeed disrupted normal activity of ASMC and CD^4+^ T cell by sponging protective miRNAs in asthma.

In addition, exposure to high-concentration ozone, an alarming phenomenon around the globe (Supplementary Tables 1 and 2), also made human beings vulnerable to asthma [16–18]. In particular, ozone not only facilitated airway smooth muscle contraction by impairing lung function and strengthening airway reactivity [19], but also encouraged abnormal inflammation of T cells through driving neutrophil multiplication [20, 21]. Notably, numerous signaling pathways relevant to immunity were altered under the influence of ozone [22], spanning from NF-κB signaling [23] to miRNA (e.g. miR-149) networks [24]. Nonetheless, few investigations were conducted to figure out if lncRNA-miRNA axes were involved in asthma development triggered by ozone.

Hence, this investigation was carried out to elucidate the association of lncRNA PVT1-led miRNA axes with ozone-induced asthma, which was conducive to clinical prevention and treatment of asthma.

## RESULTS

### Effect of ozone on airway smooth muscle function of asthma mice models

**Figure 1:**
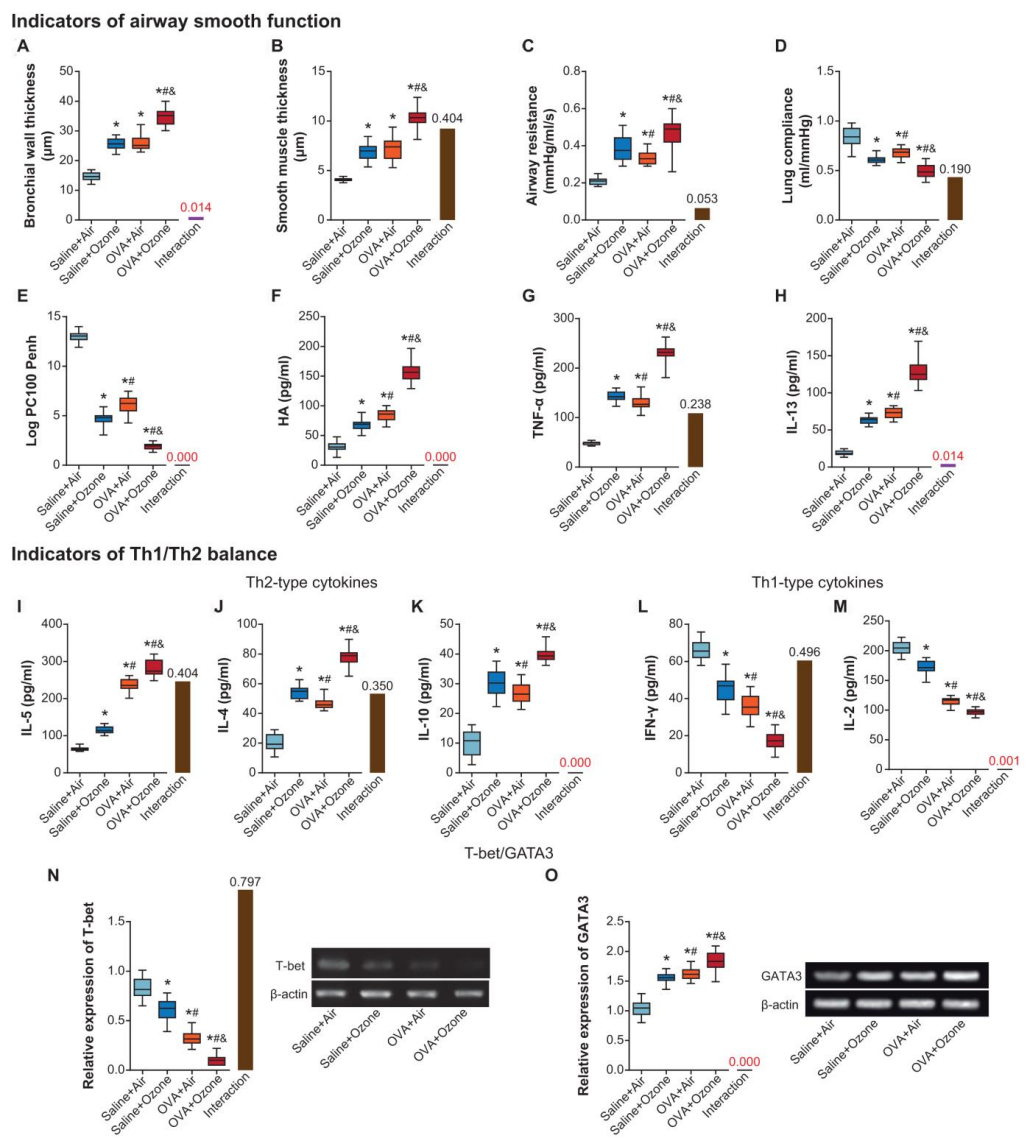
**The contribution of ozone exposure to airway smooth function and Th1/Th2 balance of asthmatic mice.** (**A**–**E**) The bronchial wall thickness (**A**), smooth muscle thickness (**B**), airway resistance (**C**), lung compliance (**D**) and Log PC100 Penh (**E**) of mice was examined among saline+air, saline+ozone, OVA+air and OVA+ozone groups. *: *P*<0.05 when compared with saline+air group, #: *P*<0.05 when compared with saline+ozone group, &: *P*<0.05 when compared with OVA+air group. (**F**–**H**) Levels of HA (**F**), TNF-α (**G**) and IL-13 (**H**) were determined within mice of saline+air, saline+ozone, OVA+air and OVA+ozone groups. *: *P*<0.05 when compared with saline+air group, #: *P*<0.05 when compared with saline+ozone group, &: *P*<0.05 when compared with OVA+air group. (**I**–**O**) The amounts of IL-5 (**I**), IL-4 (**J**), IL-10 (**K**), IFN-γ (**L**), IL-2 (**M**), T-bet (**N**) and GATA3 (**O**) were determined within mice treated by saline+air, saline+ozone, OVA+air and OVA+ozone. *: *P*<0.05 when compared with saline+air group, #: *P*<0.05 when compared with saline+ozone group, &: *P*<0.05 when compared with OVA+air group.

Bronchial wall and smooth muscle became thicker in OVA+ozone group than in saline+ozone group and OVA+air group (*P*<0.05) (Figure 1A, 1B). OVA (i.e. OVA+air group) and ozone (i.e. saline+ozone group) treatments also increased airway resistance and decreased lung compliance of mice models, as compared with saline+air group (*P*<0.05) (Figure 1C, 1D). Furthermore, ozone and OVA seemed interdependent in affecting LogPC_100_ Penh, and OVA+ozone group demonstrated lower LogPC_100_ Penh than saline+air group and OVA+air group (*P*<0.05) (Figure 1E). Hyaluronan (HA) (Figure 1F), TNF-α (Figure 1G) and IL-13 (Figure 1H) levels also reached a peak in ozone+OVA group, and they were higher in saline+ozone group and OVA+air group than in saline+air group (*P*<0.05).

### Impact of ozone on Th1/Th2 balance of asthma mice models

IL-5, IL-4 and IL-10 levels went higher in OVA+ozone group than in OVA+air group and saline+ozone group (*P*<0.05) (Figures 1I–1K), indicating that ozone and OVA engendered Th2-biased response more significantly than OVA or ozone alone. On the contrary, Th1-like cytokine levels, including IFN-γ (Figure 1L) and IL-2 (Figure 1M), were restrained in OVA+ozone group in comparison to saline+ozone group and OVA+air group (*P*<0.05). Moreover, changes of T-bet expression were consistent with that of Th1-like cytokines (Figure 1N), yet expressional variation of GATA3 followed a tendency identical to Th2-like cytokines (Figure 1O).

### Association of lncRNA PVT1 expression with airway smooth muscle function and Th1/Th2 balance of asthma mice models

**Figure 2:**
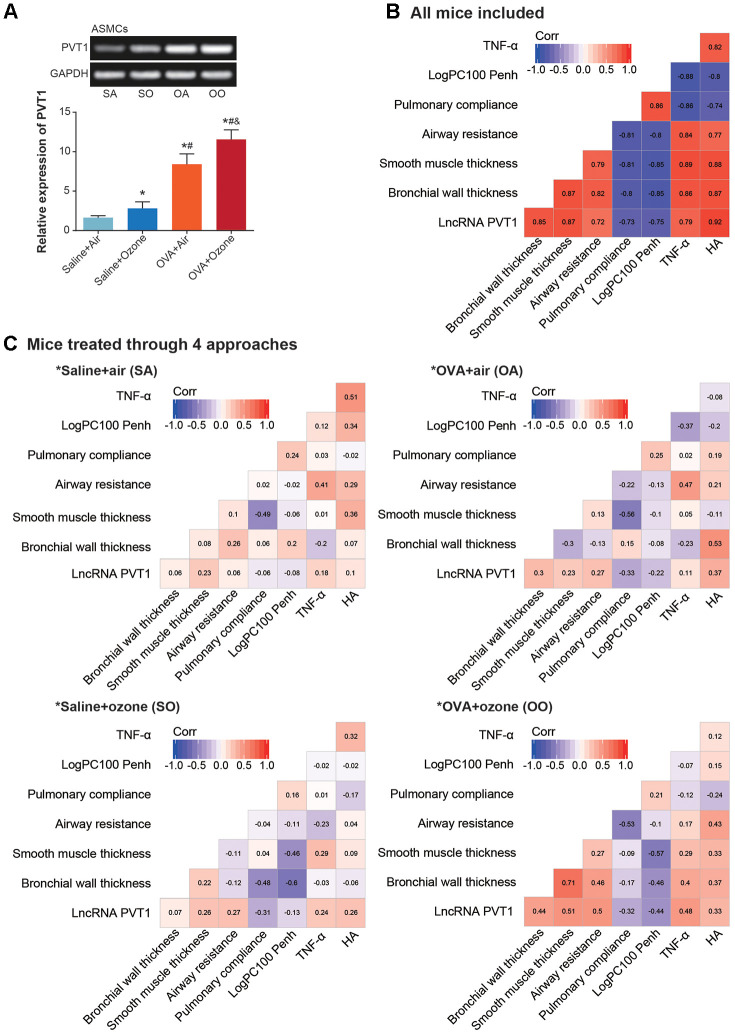
**Association of lncRNA PVT1 with airway smooth function of asthmatic mice.** (**A**) PVT1 expression was determined within ASMCs that were extracted from mice models of saline+air (SA), saline+ozone (SO), OVA+air (OA) and OVA+ozone (OO) groups. *: *P*<0.05 when compared with SA group, #: *P*<0.05 when compared with saline+ozone group, &: *P*<0.05 when compared with OVA+air group. (**B**, **C**) Correlation matrixes were generated regarding PVT1 expression and airway smooth function in all asthmatic mice (**B**) and in asthmatic mice handled through 4 approaches (**C**).

**Figure 3:**
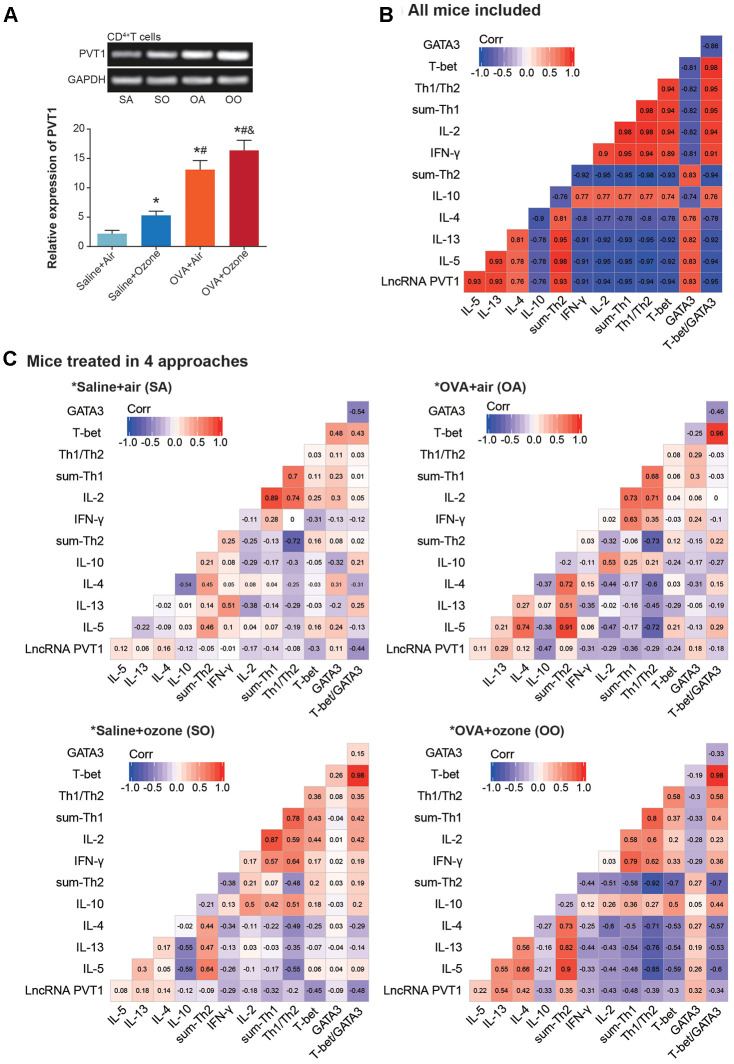
**Linkage of lncRNA PVT1 expression with Th1/Th2 balance of asthmatic mice.** (**A**) PVT1 expression was monitored within CD^4+^ T cells that were obtained from mice models of saline+air (SA), saline+ozone (SO), OVA+air (OA) and OVA+ozone (OO) groups. *: *P*<0.05 when compared with SA group, #: *P*<0.05 when compared with saline+ozone group, &: *P*<0.05 when compared with OVA+air group. (**B**, **C**) Correlation matrixes were established concerning PVT1 expression and Th1/Th2-specific cytokines in all asthmatic mice (**B**) and in asthmatic mice managed through 4 approaches (**C**).

PVT1 expression was significantly elevated in CD^4+^ T cells and ASMCs of OVA+ozone group, as compared with saline+ozone group and OVA+air group (*P*<0.05) (Figures 2A and 3A). When mice of all subgroups were considered, we found that PVT1 expression in ASMCs was significantly correlated with indicators of airway smooth muscle function, including bronchial wall thickness, smooth muscle thickness, airway resistance, pulmonary compliance, logPC100 Penh, TNF-α level and HA level (Figure 2B). On the other hand, PVT1 expression in CD^4+^ T cells was highly relevant to amount of Th1/Th2-type cytokines (Figure 3B). Intriguingly, the correlations were stronger in mice of OVA+ozone group than in mice of saline+ozone group and OVA+air group (Figures 2C and 3C), suggesting that OVA and ozone might impose additive effects on PVT1 expression in ASMCs and CD^4+^ T cells.

### Identification of miRNAs that were involved in PVT1-mediated airway smooth muscle function and Th1/Th2 balance

**Figure 4:**
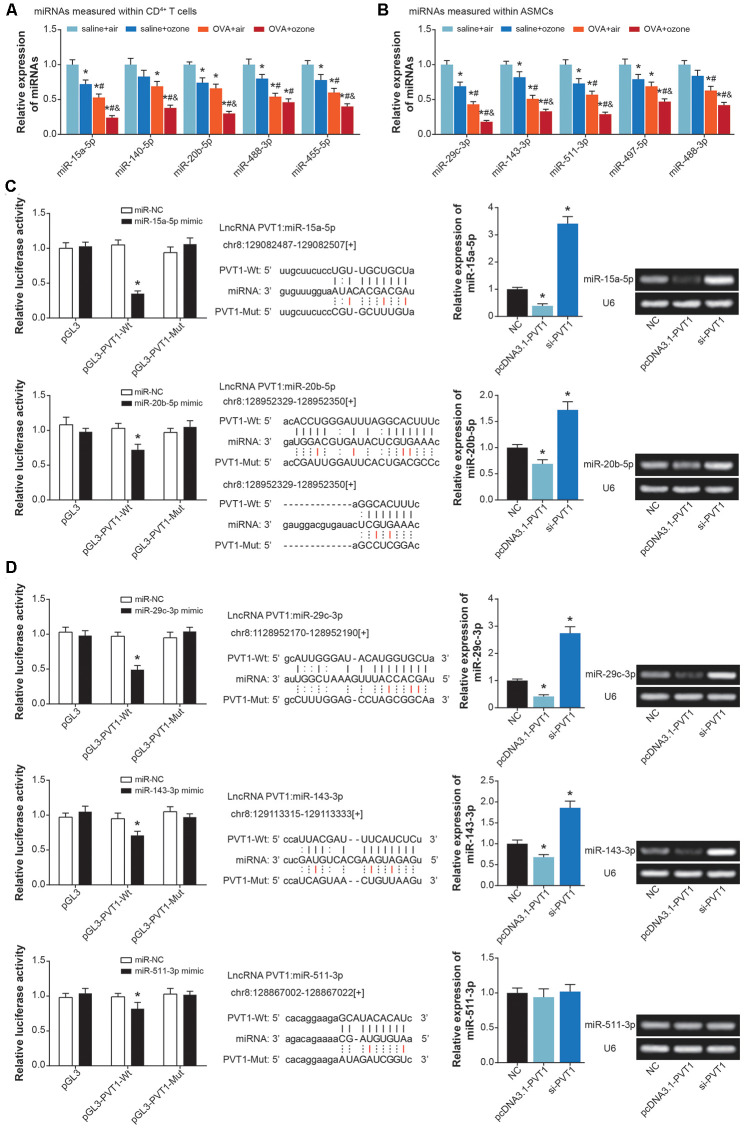
**MiRNAs potentially sponged by lncRNA PVT1 within ASMCs and CD^4+^ T cells.** (**A**) MiR-15a-5p, miR-140-5p, miR-20b-5p, miR-488-3p and miR-455-5p were differentially expressed within CD^4+^ T cells of saline+air, saline+ozone, OVA+air and OVA+ozone groups. *: *P*<0.05 when compared with SA group, #: *P*<0.05 when compared with saline+ozone group, &: *P*<0.05 when compared with OVA+air group. (**B**) Expressions of miR-29c-3p, miR-143-3p, miR-511-3p, miR-497-5p and miR-488-3p were evaluated within ASMCs of mice models among saline+air, saline+ozone, OVA+air and OVA+ozone groups. *: *P*<0.05 when compared with SA group, #: *P*<0.05 when compared with saline+ozone group, &: *P*<0.05 when compared with OVA+air group. (**C**) MiR-15a-5p and miR-20b-5p were sponged and regulated by lncRNA PVT1 in CD^4+^ T cells. (**D**) MiR-29c-3p, miR-143-3p and miR-511-3p were subjected to sponged modulation by lncRNA PVT1 in ASMCs.

MiRNAs potentially sponged by PVT1 were predicted with usage of starbase software [25] (Supplementary material), and asthma-relevant miRNAs were measured in CD^4+^ T cells and ASMCs isolated from mice models (Figure 4A and 4B, Supplementary Figure 1). It was demonstrated that miR-15a-5p, miR-140-5p, miR-20b-5p, miR-488-3p and miR-455-5p expressions were significantly down-regulated in CD^4+^ T cells of OVA- and ozone-treated mice (Figure 4A), while miR-29c-3p, miR-143-3p, miR-511-3p, miR-497-5p and miR-488-3p became lowly expressed in ASMCs of OVA/ozone-exposed mice (Figure 4B).

Furthermore, luciferase activity of CD^4+^ T cells in the miR-15a-5p/miR-20b-5p mimic+pGL3-PVT1-Wt group was decreased in comparison to miR-NC+pGL3-PVT1-Wt group and miR-15a-5p/miR-20b-5p mimic+pGL3-PVT1-Mut group (*P*<0.05) (Figure 4C). And miR-15a-5p/miR-20b-5p expression in CD^4+^ T cells was up-regulated by si-PVT1 and down-regulated by pcDNA3.1-PVT1 (*P*<0.05), suggesting that miR-15a-5p and miR-20b-5p in CD^4+^ T cells were suppressed after being sponged by PVT1. Concerning ASMCs, their luciferase activity was attenuated in the miR-29c-3p/miR-143-3p/miR-511-3p mimic+pGL3-PVT1-Wt group, when compared with miR-NC+pGL3-PVT1-Wt group and miR-29c-3p/miR-143-3p/miR-511-3p mimic+pGL3-PVT1-Mut group (*P*<0.05) (Figure 4D). And miR-29c-3p and miR-143-3p expressions were lessened by pcDNA3.1-PVT1 and yet increased by silencing of PVT1 (*P*<0.05), implying that PVT1 possibly sponged miR-29c-3p/miR-143-3p and inhibited their expression.

**Figure 5:**
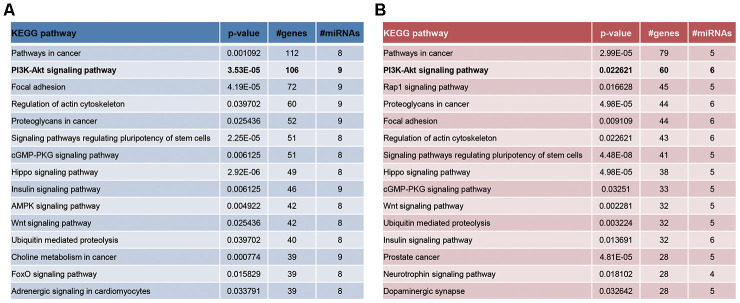
The enrichment pathways of significantly-expressed miRNAs in CD^4+^ T cells (**A**) and ASMCs (**B**) were drawn based on miRPath software.

What’s more, PI3K-Akt signaling and mTOR signaling were KEGG pathways enriched by genes targeted by significant miRNAs in CD^4+^ T cells and ASMCs (Figure 5), which was draw from miRPath software [26]. Interestingly, the PI3K-Akt-mTOR signaling counted much in lung inflammation and airway remodeling of asthma [27–32], and they were involved in propelling ozone-stimulated oxidative stress [33, 34]. It was thus speculated that PVT1-miRNA axis might participate in ozone-induced asthma development by activating PI3K/Akt/mTOR signaling.

### PVT1 sponged miR-29c-3p to facilitate proliferation and depress apoptosis of ASMCs

**Figure 6:**
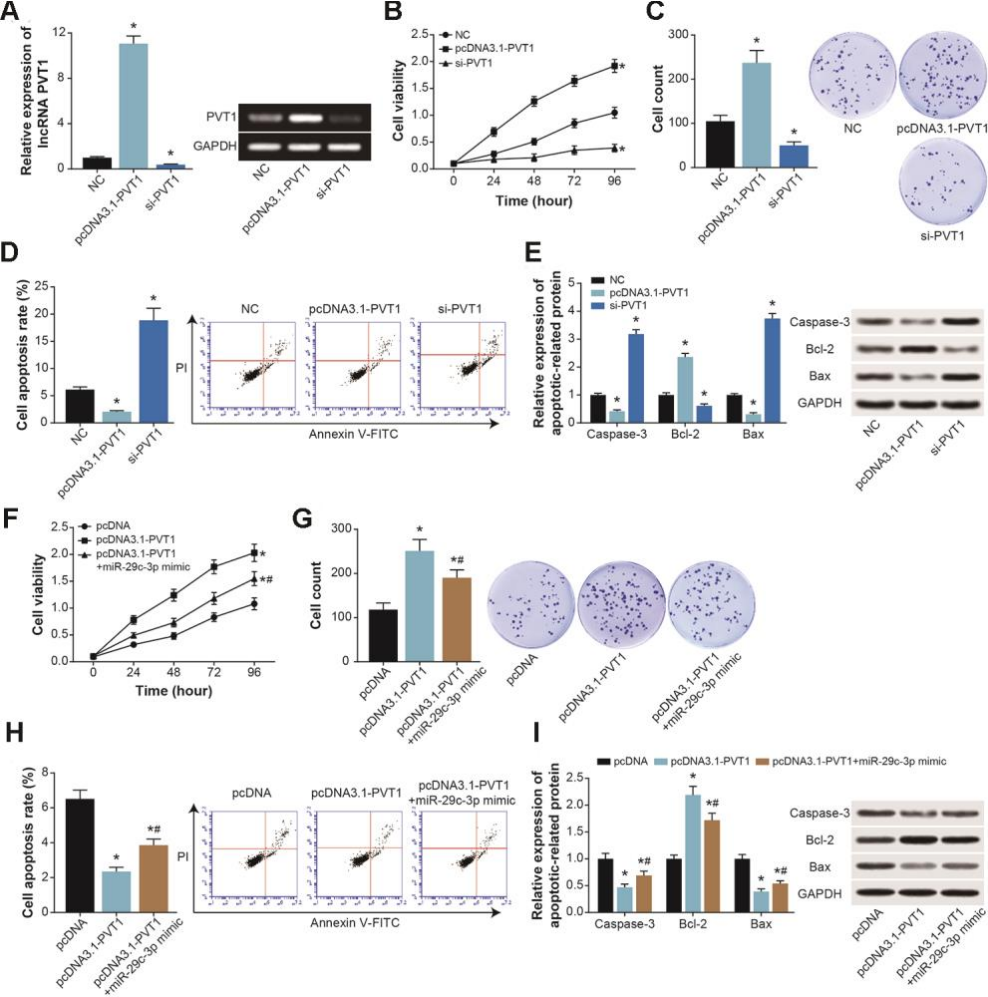
**The effects of PVT1 and miR-29c-3p on proliferation and apoptosis of ASMCs.** (**A**) PVT1 expression in ASMCs was determined after transfection of pcDNA-PVT1 and si-PVT1. *: *P*<0.05 when compared with NC group. (**B**–**D**) The viability (**B**), proliferation (**C**) and apoptosis (**D**) of ASMCs were compared after transfections of pcDNA-PVT1 and si-PVT1. *: *P*<0.05 when compared with NC group. (**E**) The expressions of apoptotic proteins within ASMCs were compared under treatments of pcDNA-PVT1and si-PVT1. *: *P*<0.05 when compared with NC group. (**F**–**H**) The viability (**F**), proliferation (**G**) and apoptosis (**H**) of ASMCs were evaluated among pcDNA3.1, pcDNA3.1-PVT1 and pcDNA3.1-PVT1+miR-29c-3p mimic groups. *: *P*<0.05 when compared with pcDNA3.1 group, #: *P*<0.05 when compared with pcDNA3.1-PVT1 group. (**I**) Expressions of apoptins were measured within ASMCs of pcDNA3.1, pcDNA3.1-PVT1 and pcDNA3.1-PVT1+miR-29c-3p mimic groups. *: *P*<0.05 when compared with pcDNA3.1 group, #: *P*<0.05 when compared with pcDNA3.1-PVT1 group.

PVT1 expression in ASMC was elevated dramatically after transfection of pcDNA3.1-PVT1, yet its expression revealed a drop when si-PVT1 was transfected (*P*<0.05) (Figure 6A). Moreover, pcDNA3.1-PVT1 strengthened viability and proliferation, yet hindered apoptosis of ASMCs (*P*<0.05) (Figure 6B–6D). By contrast, viability and proliferation of ASMCs were weakened, and ASMC apoptosis was accelerated by si-PVT1 (*P*<0.05). Furthermore, bax/caspase-3 expressions were down-regulated, and bcl-2 expression was up-regulated in cases of pcDNA3.1-PVT1 transfection (*P*<0.05), whereas si-PVT1 group exhibited higher bax/caspase-3 expressions and lower bcl-2 expression than si-NC group (*P*<0.05) (Figure 6E). It was noteworthy that viability and proliferation of ASMCs were impaired in the pcDNA3.1-PVT1+miR-29c-3p mimic group in comparison to pcDNA3.1-PVT1 group (*P*<0.05) (Figure 6F and 6G), yet ASMC apoptosis was boosted in the pcDNA3.1-PVT1+miR-29c-3p mimic group as relative to pcDNA3.1-PVT1 group (*P*<0.05) (Figure 6H). Expressions of caspase-3 and bax were also raised, along with decreased expression of bcl-2, in pcDNA3.1-PVT1+miR-29c-3p mimic group, when compared with pcDNA3.1-PVT1 group (*P*<0.05) (Figure 6I).

### PI3K/AKT signaling reversed inhibition of miR-29c-3p on ASMC viability, proliferation and apoptosis

**Figure 7:**
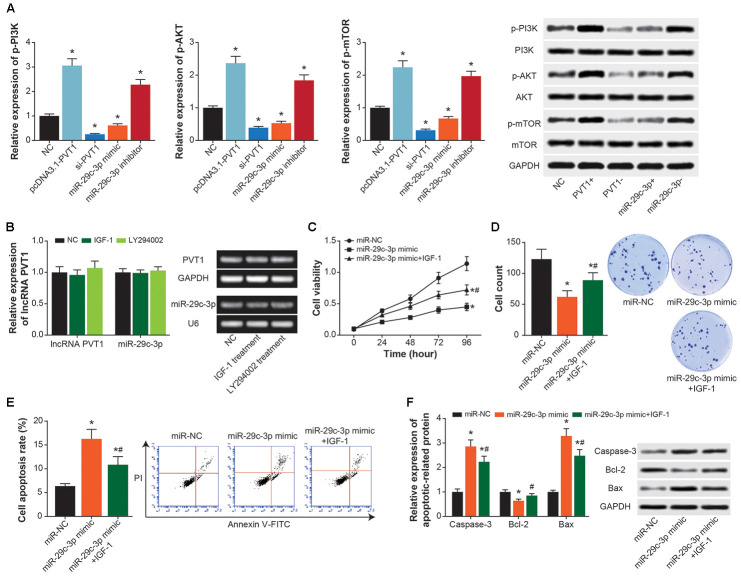
**PI3K/AKT/mTOR signaling mediated the impact of PVT1/miR-29c-3p axis on ASMC activity.** (**A**) Expressions of p-PI3K, PI3K, p-AKT, AKT, p-mTOR and mTOR were determined within ASMCs that were treated by none, pcDNA3.1-PVT1, si-PVT1, miR-29c-3p mimic and miR-29c-3p inhibitor. *: *P*<0.05 when compared with NC group. (**B**) Expressions of PVT1 and miR-29c-3p were assessed in ASMCs managed by IGF-1 and LY294002. *: *P*<0.05 when compared with NC group. (**C**–**E**) Viability (**C**), proliferation (**D**) and apoptosis (**E**) of ASMC were appraised among miR-NC, miR-29c-3p mimic and miR-29c-3p mimic+IGF-1 groups. *: *P*<0.05 when compared with miR-NC group, #: *P*<0.05 when compared with miR-29c-3p mimic group. (**F**) Expressions of apoptins were measured within ASMCs of miR-NC, miR-29c-3p mimic and miR-29c-3p mimic+IGF-1 groups. *: *P*<0.05 when compared with miR-NC group, #: *P*<0.05 when compared with miR-29c-3p mimic group.

Expressions of p-PI3K, p-AKT and p-mTOR in ASMCs were heightened by pcDNA3.1-PVT1 or miR-29c-3p inhibitor (*P*<0.05), and they were suppressed by si-PVT1 and miR-29c-3p mimic (*P*<0.05) (Figure 7A). However, IGF-1 (i.e. activator of PI3K/AKT signaling) and LY294002 (i.e. inhibitor of PI3K/AKT signaling) treatments failed to alter PVT1 and miR-29c-3p expressions in ASMCs (*P*>0.05) (Figure 7B). In addition, proliferation and viability of ASMCs were significantly motivated in the miR-29c-3p mimic+IGF-1 group as compared with miR-29c-3p mimic group (*P*<0.05) (Figure 7C and 7D), and ASMCs in the miR-29c-3p mimic+IGF-1 group were less prone to apoptosis than ASMCs in the miR-29c-3p mimic group (*P*<0.05) (Figure 7E and 7F).

### PVT1/miR-15a-5p axis promoted Th1/Th2 imbalance of CD^4+^T cells by activation of PI3K-AKT signaling

**Figure 8:**
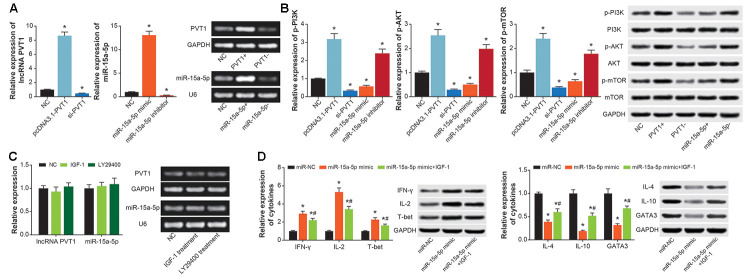
**PI3K/AKT/mTOR signaling was implicated in the contribution of PVT1/miR-15a-5p axis to Th1/Th2 balance in CD^4+^ T cells.** (**A**) PVT1 expression was determined after transfection of pcDNA3.1-PVT1 and si-PVT1, and miR-15a-5p level was compared in CD^4+^ T cells transfected by miR-15a-5p mimic and inhibitor. *: *P*<0.05 when compared with miR-NC group. (**B**) The p-PI3K, PI3K, p-AKT, AKT, p-mTOR and mTOR expressions in CD^4+^ T cells were figured out among NC, pcDNA3.1-PVT1, si-PVT1, miR-15a-5p mimic and miR-15a-5p inhibitor groups. *: *P*<0.05 when compared with NC group. (**C**) PVT1 and miR-15a-5p expressions were obtained from CD^4+^ T managed by IGF-1 and LY294002. *: *P*<0.05 when compared with NC group. (**D**) The levels of cytokines relevant to Th1/Th2 imbalance were determined within CD^4+^ T cells among miR-NC, miR-15a-5p mimic and miR-15a-5p mimic+IGF-1 groups. *: *P*<0.05 when compared with miR-NC group, #: *P*<0.05 when compared with miR-15a-5p mimic group.

PVT1 expression was significantly increased in CD^4+^ T cells transfected by pcDNA3.1-PVT1 (*P*<0.05), and was decreased by transfection of si-PVT1 (*P*<0.05) (Figure 8A). MiR-15a-5p expression in CD^4+^ T cells was enhanced by miR-15a-5p mimic (*P*<0.05), and was diminished by miR-15a-5p inhibitor (*P*<0.05) (Figure 8A). Moreover, pcDNA3.1-PVT1 and miR-15a-5p inhibitor were found to activate p-PI3K, p-AKT and p-mTOR in CD^4+^ T cells (*P*<0.05), which were deactivated by si-PVT1 and miR-15a-5p mimic (*P*<0.05) (Figure 8B). Nevertheless, expressions of PVT1 and miR-15a-5p in CD^4+^ T cells were almost unchanged by IGF-1 and LY29400, when compared with untreated CD^4+^ T cells (*P*>0.05) (Figure 8C). Furthermore, CD^4+^ T cells of miR-15a-5p mimic+IGF-1 group engendered lower protein levels of IFN-γ, IL-2 and T-bet and higher protein levels of IL-4, IL-10 and GATA3 than CD^4+^ T cells of miR-15a-5p mimic group (*P*<0.05) (Figure 8D).

### Diagnostic performance of PVT1 in asthma

Higher serum levels of TNF-α, IL-13, IL-4, IL-10, hs-CRP and FeNO were determined in asthma patients than in healthy volunteers (*P*<0.05) (Supplementary Table 3). The cytokine levels also demonstrated an upward trend in patients with acute asthma when compared with patients in the remission stage of asthma (*P*<0.05). Conversely, asthma patients were associated with smaller FEV1 and lower FEV1/FVC ratio than healthy controls (*P*<0.05), and both FEV1 and FEV1/FVC ratio were lessened in patients with acute asthma as compared with asthma patients in remission stage (*P*<0.05). Serum level of PVT1 also went up significantly in asthma patients when compared with healthy controls (*P*<0.05), and patients with acute asthma revealed higher PVT1 expression than asthma patients in remission stage (*P*<0.05) (Supplementary Figure 2A). It seemed that PVT1 was promising in diagnosis of asthma (AUC=0.909) (Supplementary Table 4, Supplementary Figure 2B) and in differentiating patients with acute asthma from asthma patients in remission stage (AUC=0.705) (Supplementary Table 4, Supplementary Figure 2C).

## DISCUSSION

Here we attempted to uncover whether lncRNA PVT1-miRNA axis was implicated in the pathogenesis of ozone-induced asthma. Firstly, mice models of asthma were established [35], and it was intriguing to notice that acute exposure to ozone aggravated asthma symptoms in OVA-exposed mice models [36] (Figure 1). Specifically, airway wall and airway smooth muscle were thickened (Figure 1A and 1B), along with intensified airway stenosis, in ozone-exposed asthmatic mice (Figure 1C and 1D), suggesting that ozone drove airway remodeling and suppressed pulmonary compliance of asthmatic mice. Changes of log100 Penh value (Figure 1E), determined by means of pneumotachograph of whole-body plethysmography [37], reflected that airway responsiveness of asthmatic mice was reduced after exposure to ozone, and the noticeable increase in levels of TNF-α and HA (Figure 1F and 1G) suggested that ASMC proliferation in OVA-exposed mice was promoted by ozone [38–41]. In addition to airway function controlled by ASMC, Th1/Th2 balance manipulated by CD^4+^ T cells was also aggravated by OVA exposure and ozone [10, 42, 43], detailed as that ozone increased Th2/Th1 ratio (Figure 1I-1M) and decreased T-bet/GATA3 ratio (Figure 1N and 1O) in OVA-exposed mice.

There have been evidence that ozone triggered asthma onset by inducing airway responsiveness and airway inflammation [44–46]. The proponents held that oxygen free radicals generated by ozone attacked intra-membranous polyunsaturated fatty acid and triggered oxidative damage in organisms [47–49], which finally worsened inflammation in airway [50, 51]. However, a contradiction existed that prevalence of asthma was disproportionate to worldwide concentration of ozone (https://www.stateofglobalair.org/data/#/air/map) (Supplementary Tables 1 and 2) [52]. We speculated that distinctions in detecting instrument, operation step and measurement standard might blur the actual impact of ozone on asthma onset. Other contributors to asthma, such as family history and abrupt climate change, could also confuse the internal association of ozone with asthma development. Of note, PVT1, which contained a genomic region indicative of high cancer risk [53, 54], was conjectured to involve in ozone-caused asthma progression, allowing for that PVT1 expression was strongly correlated with ASMC function and Th1/Th2 balance of ozone-treated asthma mice models (Figures 2, 3). This might be ascribed to the strength of PVT1 in promoting ASMC multiplication and in boosting secretion of Th2-type cytokines as opposed to Th1-type cytokines by CD^4+^ T cells.

Founded on the ceRNA theory, we suspected that PVT1 probably urged asthma progression by sponging miR-15a-5p and miR-29c-3p, two protective miRNAs in asthma [55]. The miR-15a-5p was documented to hinder production of inflammatory chemokines (e.g. IL-10) [56], and to prevent onset of T cell-relevant diseases [57]. Here PVT1 was speculated to promote Th2-orineted inflammation in CD^4+^ T cells by resisting function of miR-15a-5p (Figure 4C, Figure 8D), which was a highlight of this study. With regard to miR-29c-3p, which was down-regulated in asthma children [58], we found it effective in reversing the contribution of PVT1 to ASMC proliferation and viability (Figure 6F–6I). Despite difference in cell type, miR-29c-3p was capable of holding up proliferation, invasion and metastasis of tumor cells [59, 60], including lung cancer, hepatic carcinoma, gastric cancer, glioma and leukemia [61–63], And this might explain why miR-29c-3p prevented ASMC abnormality in asthma from the molecular side.

In addition, PI3K/Akt/mTOR signaling was a widely-recognized driver of airway inflammation and airway remodeling underlying asthma etiology. For instance, PI3K was able to reinforce inflammation response mediated by eosinophilic granulocyte, T cell, mastocyte and neutrophil, which stayed core to asthma onset [64]. Th2-centric inflammation response was also promoted by PI3K [65], and Akt activation tended to guide differentiation of Th cells into Th2-like CD^4+^ T cells [66]. Furthermore, PI3K-dependent P70S6K activation was implicated in controlling mitogen-stimulated response of ASMC [67], and PI3K/Akt/mTOR signaling was capable of worsening airway remodeling by stimulating ASMC proliferation [68–72]. Our study newly introduced that PI3K/Akt/mTOR signaling not merely diminished the effect of miR-15a-5p on Th1/Th2 balance in CD^4+^ T cells (Figure 8D), but also undermined the impact of miR-29c-3p on ASMC proliferation (Figure 7C–7F), which expanded knowledge about asthma pathogenesis.

## CONCLUSIONS

**Figure 9:**
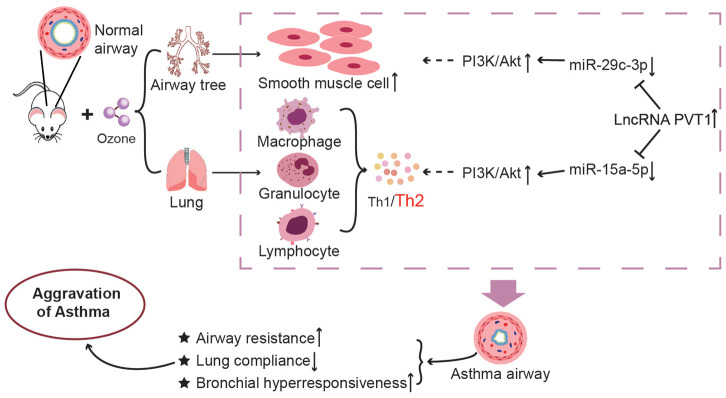
**The mechanism diagram about impacts of lncRNA PVT1–centered miRNA networks and ozone on asthmatic progression.** It was revealed that exposure to ozone impacted Th1/Th2 imbalance of CD^4+^ T cells by regulating lncRNA PVT1-miR-15a-5p-PI3K/AKT/mTOR axis and apoptosis of ASMCs through modifying lncRNA PVT1-miR-29c-3p- PI3K/AKT/mTOR axis.

In conclusion, this investigation tentatively verified that ozone exacerbated asthma development by activating PVT1-miR-15a-5p/miR-29c-3p signaling, which motivated Th1/Th2 imbalance of CD^4+^ T cells and urged excessive proliferation of ASMCs (Figure 9). Nonetheless, although cell models and animal models were established, clinical evidence was insufficient to support this hypothesis, which necessitated more convincing evidence. Secondly, genes that encoded oxidative/non-oxidative enzymes were not detected, so impacts of ozone and OVA on in-vivo oxidative stress could not be verified. Last but not the least, ozone level applied here was above the concentration of natural exposure and also exceeded the concentration which induced asthma onset (i.e. 0.06 ppm) [73]. It might be better if later researches were designed to tally with practical settings.

## MATERIALS AND METHODS

### Establishment of mice models with acute asthma

A total of 168 female BALB/C mice, aged 8-10 weeks old and weighing around 20 g, were purchased from Shanghai Laboratory Animal Center (Shanghai, China). The mice, exposed under a 12h/12h light/dark cycle, were housed in individually ventilated cages that were controlled at a 24° C and in 60% humidity. We divided the mice into saline+air group (n=42), OVA+air group (n=42), saline+ozone (n=42) group and OVA+ozone (n=42) group, and their treatments were particularized in Supplementary Figure 3.

### Assessment of airway resistance and pulmonary compliance in asthma mice models

After being anesthetized by 80 mg/kg pentobarbital for 10 min, trachea of mice models was separated by cutting cervical region and peeling upper airway. The trachea was cut between the 2^nd^ and the 3^rd^ cricoid cartilage, and a cannula (diameter: 0.9 mm) was inserted into the airway until a depth of 3-4 mm. Then a knot was tied to fix the 4^th^ and the 5^th^ trachea cartilage ring, and cannula was connected to animal ventilator (model: SAR-830, CWE corporation, USA). Airway resistance (R) and pulmonary compliance of mice models were automatically calculated by MF Lab software (version 3.01).

### Evaluation of bronchial reactivity and airway hyper-responsiveness (AHR) in asthma mice models

Bronchial reactivity of mice models was monitored using non-invasive whole-body plethysmograph (model: FinePointe™NAM, Bucxo, USA). Basic enhanced pause (Penh) value and Penh values under treatments of 0, 1.56, 3.12, 6.25, 12.5, 25 and 50g/L methacholine (MCH) were detected [74], and airway reactivity was calculated according to the formula of $\frac{\text{average Penh value within }7\text{ min }}{\text{basal }Penh\;value}\times \;100\%$. AHR (i.e. Log PC_100_) was equivalent to the logarithm of MCH concentration that mice required to achieve 2 folds of their basal airway responsiveness.

### Measurement of bronchoalveolar cytokines in asthma mice models

A polyethylene catheter (diameter: 1.0 mm) was inserted into the slot which was cut between the 2^nd^ and the 3^rd^ cartilaginous rings of mice models. Then icy PBS was injected into lung of mice models with a syringe (model: 1 ml), and 80% of the lavage fluid was recycled. After centrifugation at 7500×g for 5 min, IL-5, IL-13, TNF-α, HA, IFN-γ, IL-2, IL-4 and IL-10 levels in the supernatant were determined with ELISA kits (R&D Systems, USA).

### Appraisal of bronchial wall/smooth muscle thickness in asthma mice models

Lung hilum cut from mice models was immersed within 4% paraformaldehyde, and 6 h later they were immersed in 70% ethanol and embedded by paraffin. Slices stained by hemotoxylin-eosin (HE) were observed under 200× light microscope, and bronchial walls with complete structure were selected. Circumferential diameter of bronchial basement membrane (Pbm), total area of bronchial wall (Wat) and area of bronchial smooth muscle (Wam) were gauged aided by IPP 6.0 image analysis software. Wat/Pbm ratio and Wam/Pbm ratio were, respectively, indicative of bronchial wall thicknesses and smooth muscle thicknesses.

### Isolation of CD^4+^ T cells and ASMCs from asthma mice models

### Isolation of CD^4+^ T cells from spleen of mice models

After centrifugation at the spend of 2000 r/min, monocytes were re-suspended within 5 ml RPMI1640 medium which contained 10% FCS (Gibco, USA). Monocytes adjusted to the concentration of 1×10^8^/ml were incubated with 1 ml rat anti-mouse CD4 antibody (eBioscience, USA) for 15 min at room temperature. Then cell suspension that flew through separation column (R&D Systems, USA) was collected, until liquid effluent became clear.

### Extraction of ASMCs from trachea of mice models

Tracheal of mice models was separated, and their bronchus was vertically dissected and cut into tissue blocks sized as 1 mm^3^. The cells were then cultivated in 5% CO_2_ at 37° C, until digestion by 0.25% trypsin on the 10^th^ day. ASMCs were confirmed by performing α-actin immune-cytochemical staining.

### Cell transfection

With assistance of Lipofectamine® RNAiMAX transfection kit (Thermo Fisher Scientific, USA), si-PVT1 (Genepharma, China), si-NC, pcDNA3.1-PVT1 (Genepharma, China), miR-15a-5p mimic and miR-15a-5p inhibitor (Ribobio, China) were, respectively, transfected into CD^4+^ T cells. On the other hand, si-PVT1, pcDNA3.1-PVT1, miR-29c-3p mimic and miR-29c-3p inhibitor (Genepharma, China) were transfected into ASMCs, respectively. ASMCs and CD^4+^ T cells were cultivated within Opti-MEM^®^ reduced serum medium (Thermo Fisher Scientific, USA), until they grew to > 70% confluency.

### Cell treatment

CD^4+^ T cells and ASMCs were treated by insulin-like growth factor (IGF-1, Peprotech, USA) at the final concentration of 1 μmol/L or LY294002 (Selleck, USA) at the final concentration of 50 μmol/L.

### Evaluation of ASMC activity

### CCK-8 assay

ASMCs inoculated at the concentration of 1×10^4^/ml were cultivated until they became adherent to the plate wall. Then ASMCs of each well were incubated by 10 μl CCK 8 reagent (DOJINDO, Japan) for 4 h. Absorbance (A) of ASMCs was measured on the enzyme-linked immunosorbent assay system (model: ElX800, BIO-TECH, USA) at the wavelength of 450 nm.

### Colony formation assay

ASMCs at the density of 4×10^3^/well were cultured in 6-well plates for 9 days, and then they were fixed by 4% paraformaldehyde for 15 min and dyed by 0.1 % crystal violet for 20 min. Photographs were taken after air-dry of ASMCs.

### Cell apoptosis assay

When ASMCs (concentration: 1 × 10^5^/well) grew to cover 70% of cell plate, they were mixed by 10 μl Annexin V-FITC (Sigma, USA) and 5 μl propidium iodide (PI) (Sigma, USA). After 15-min culture in the darkness, percentage of apoptotic ASMCs was assessed on flow cytometer (model: FACSAria, BD, USA).

### Collection of blood samples from asthma patients

One hundred and forty-seven patients with asthma were recruited from The First Hospital of Shanxi Medical University. The asthma patients were all in accordance with diagnostic criteria formulated by Chinese Society of Respiratory Medicine [75], including 85 patients at the acute stage of asthma and 62 asthma patients in remission stage. Asthma patients in remission stage should not show any acute asthma symptoms in the past ≥ 1 month, and patients with acute asthma were given glucocorticoids-/bronchodilators-based treatments. Meanwhile, 46 healthy volunteers without any history of allergic diseases were incorporated, and they should not suffer from any infectious diseases for the past 2 months. All the participants have signed informed consents, and this program was approved by The First Hospital of Shanxi Medical University and the ethics committee of The First Hospital of Shanxi Medical University. Around 4 ml venous blood was taken from each subject, which was reserved at -80° C for PVT1 detection with RT-PCR.

### Western blotting

Concentration of total proteins, extracted from tissues and cells with RIPA buffer, was measured with BCA kit (Beyotime, China). After undergoing 10% sodium dodecyl sulfate-polyacrylamide gel electrophoresis (SDS-PAGE), protein samples were electrically transferred onto polyvinylidene fluoride (PVDF) membrane for 3 h. Afterwards, the samples were blocked by 5% skim milk for 1 h, followed by incubation with primary antibodies (rabbit anti-mouse, Abcam, USA) against caspase-3 (1: 500, Catalog No.: ab13847, Abcam), Bcl-2 (1: 2000, Catalog No.: ab196495, Abcam), Bax (1: 3000, Catalog No.: ab32503, Abcam), p-PI3K (1:1000, Catalog No.:17366, Cell Signaling Technology), PI3K (1:1000, Catalog No.:4292, Cell Signaling Technology), p-AKT (1:2000, Catalog No.: 4060, Cell Signaling Technology), AKT (1:1000, Catalog No.:4691, Cell Signaling Technology), p-mTOR (1:1000, Catalog No.:5536, Cell Signaling Technology), mTOR (1:1000, Catalog No.:2972, Cell Signaling Technology), IFN-γ (1: 3000, Catalog No.: ab171081, Abcam), IL-2 (1:500, Catalog No.: ab180780, Abcam), T-bet (1:1000, Catalog No.: ab154058, Abcam), IL-4 (1:1000, Catalog No.: ab9811, Abcam), IL-10 (1:1000, Catalog No.: ab189392, Abcam), GATA3 (1:1000, Catalog No.: ab182747, Abcam), GAPDH (1: 10000, Catalog No.: ab181603, Abcam). On the next day, the products were incubated by horseradish peroxidase (HRP)-labeled secondary antibody (goat anti-rabbit, 1: 10000, Catalog No.: ab97080, Abcam, USA) at room temperature for 1 h. Finally, development was accomplished with aid of Immobilon^TM^ Western Chemiluminescent kit (Merck Millipore, USA), and proteins were quantified utilizing image-pro plus 5.0 software.

### Quantitative real-time polymerase chain reaction (qRT-PCR)

Total RNAs were extracted from tissues, cells and blood samples utilizing RNAiso Plus reagent (TakaRa, Japan), and integrity of the RNAs was confirmed by agarose gel electrophoresis. The RNAs were reversely transcribed into cDNAs on the strength of SYBR green-based qRT-PCR kit (Invitrogen, USA). With primers (Table 1 and Supplementary Table 5) designed and synthesized by Genepharma (China), cDNAs were amplified by PCR under conditions of: 1) 95° C for 10 min, and 2) 40 cycles of 95° C for 15 s and 60° C for 1 min. Relative expressions of genes were calculated in accordance with 2^- ΔΔCt^ method.

**Table 1:** The sequences of the primers for the amplification used by real-time PCR.

**Subject**	**Primers (5'-3')**	
	**Forward**	**Reverse**
GATA3	CGAGAAAGAGTGCCTCAAGTACC	GAAGTCCTCCAGTGAGTCATGC
T-bet	GTGACCCAGATGATTGTGCT	GGTTGGGTAGGAGAGGAGAG
β-actin	TGGGTCAGAAGGATTCCTAT	ATGAGGTAGTCAGTCAGGTCC
PVT1	TGAGAACTGTCCTTACGTGACC	AGAGCACCAAGACTGGCTCT
GAPDH	CGTGTTCCTACCCCCAATGT	TGTCATACTTGGCAGGTTTCT
miR-15a-5p	ATCCAGTGCGTGTCGTG	TGCTTAGCAGCACATAATG
miR-29c-3p	GCCTAGCACCATTTGAAATCG	GTGCAGGGTCCGAGGT
U6	CTCGCTTCGGCAGCACA	ACGCTTCACGAATTTGCGT

### Dual-luciferase reporter gene assay

PVT1 fragments that incorporated binding sites with miR-15a-5p/miR-140-5p/miR-20b-5p/miR-488-3p/miR-455-5p were, respectively, amplified by PCR, so that PVT1-wide type (Wt) fragments corresponding to each miRNA were produced. On the other hand, PVT1-mutant type (Mut) fragments were generated similarly, except that binding sites with miR-15a-5p/miR-140-5p/miR-20b-5p/miR-488-3p/miR-455-5p were mutated in PVT1 fragments. Then PVT1 fragments were inserted into pGL3-Promoter plasmid vector (Promega, USA), so that pGL3-PVT1-Wt and pGL3-PVT1-Mut for each miRNA were constructed. Subsequently, miR-15a-5p mimic/miR-140-5p mimic/miR-20b-5p mimic/miR-488-3p mimic/miR-455-5p mimic/miR-NC (Genepharma, China) were co-transfected with pGL3-PVT1-Wt, pGL3-PVT1-Mut or pRL-TK plasmid (Promega, USA) into CD^4+^ T cells, and miR-29c-3p mimic/miR-143-3p mimic/miR-511-3p mimic/miR-497-5p mimic/miR-488-3p mimic/miR-NC (Genepharma, China) were co-transfected with pGL3-PVT1-Wt, pGL3-PVT1-Mut or pRL-TK plasmid into ASMCs. After 12-h incubation in 5% CO_2_ at 37° C, firefly luciferase activity and renin luciferase activity of CD^4+^ T cells and ASMCs were monitored on the microplate reader (BioTek, USA), in accordance with instructions of dual luciferase reporter gene detection kit (Promega, USA).

### Statistical analyses

All the data were statistically analyzed by SPSS19.0 software. Quantitative data, presented as mean ± standard deviation, were compared with student’s t test or one-way analysis of variance (ANOVA). Interaction of ozone and OVA on airway smooth muscle function and Th1/Th2 cytokine level were appraised by 2×2 factorial design, and correlation matrix was established utilizing “ggcorrplot” package of R studio software (http://www.rproject.org). It was statistically significant if *P* was smaller than 0.05.

### Ethics approval

All these operations and experimental process have been approved by the experimental animal ethics committee of The First Hospital of Shanxi Medical University.

## Supplementary Material

Supplementary Figures

Supplementary Tables

Supplementary Material 1
