# Supplementary material for: Exposure to ozone impacted Th1/Th2 imbalance of CD4+ T cells and apoptosis of ASMCs underlying asthmatic progression by activating lncRNA PVT1-miR-15a-5p/miR-29c-3p signaling
Source: Aging (Albany NY). 2020 Nov 20;12(24):25229–55. doi: 10.18632/aging.104124 (PMC7803560; doi:10.18632/aging.104124)
Supplement: Supplementary Figures [file aging-12-104124-s001.pdf]

SUPPLEMENTARY FIGURES

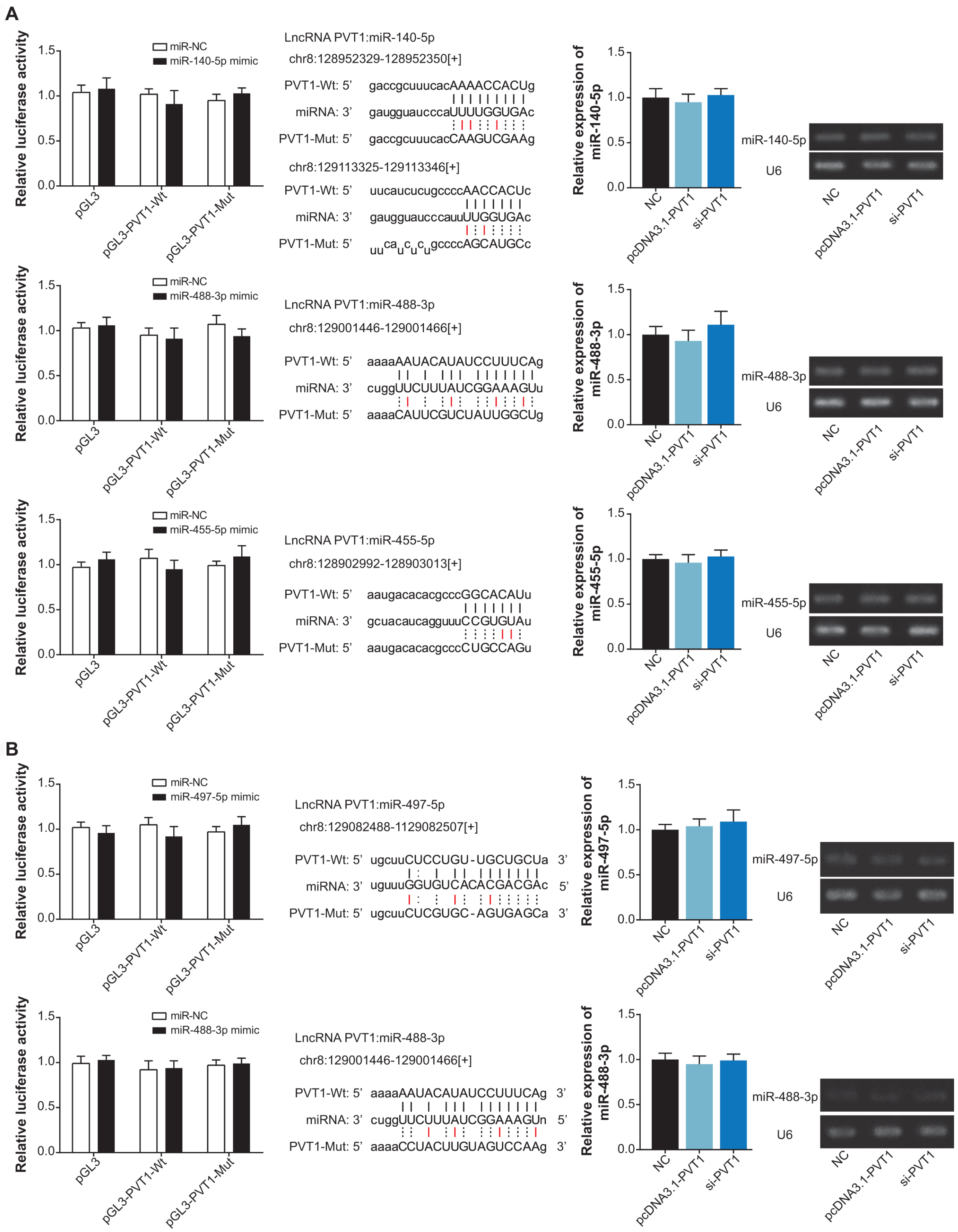

Supplementary Figure 1. MiRNAs failed to be sponged by lncRNA PVT1 within CD^{4+} T cells (**A**) and ASMCs (**B**).

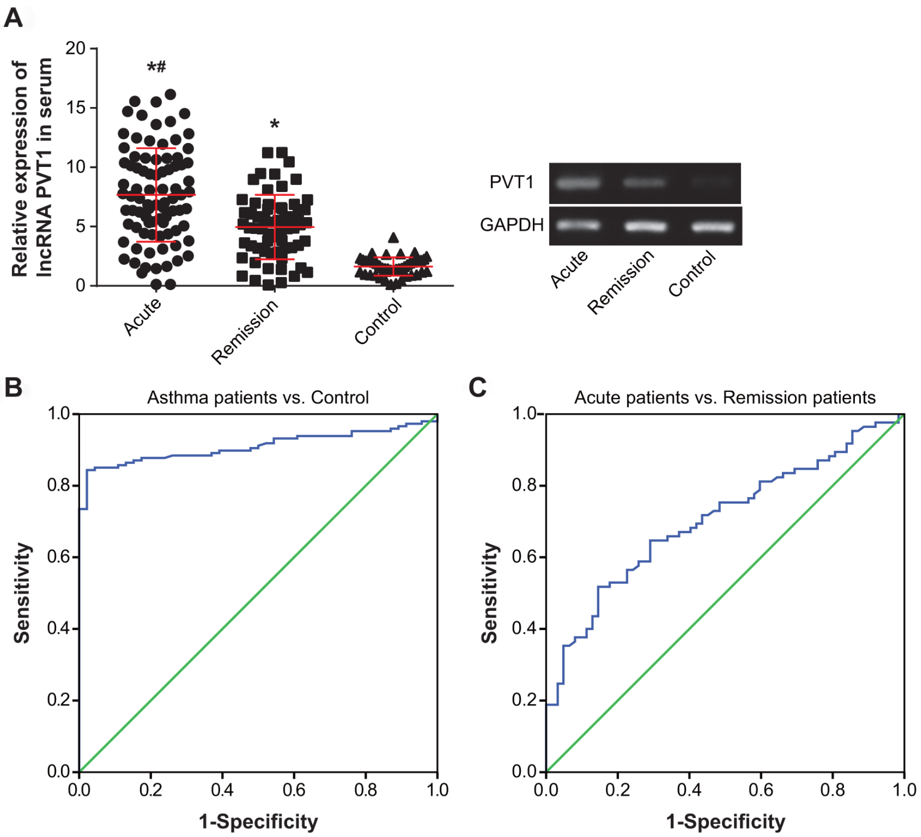

Supplementary Figure 2. Diagnostic performance of lncRNA PVT1 for asthma. (**A**) Serum level of lncRNA PVT1 was determined among patients at the acute stage of asthma, asthma patients in remission stage and healthy volunteers. (**B**) ROC curve was plotted to estimate the role of lncRNA PVT1 in diagnosis of asthma. (**C**) Serum level of lncRNA PVT1 was powerful in separating patients at the acute stage of asthma from asthma patients in remission stage.

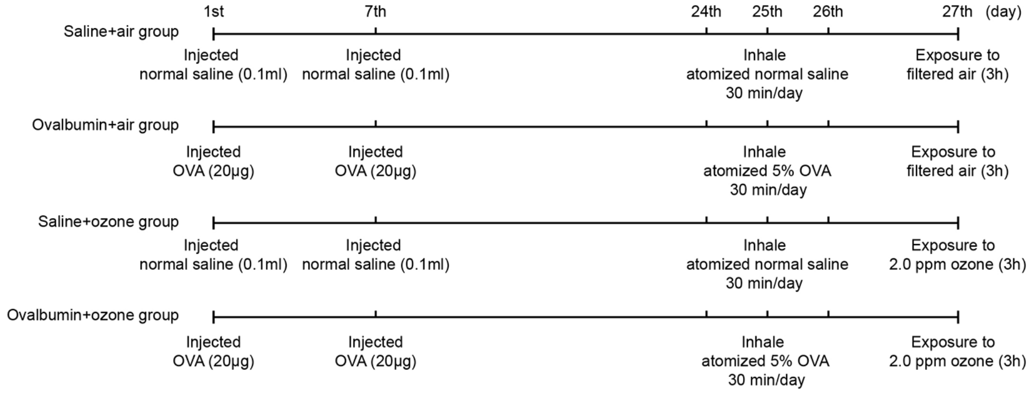

Supplementary Figure 3. The treatment scheme for establishing mice models. 1) mice in the saline+air group (control group) were intraperitoneally injected with 0.1 ml normal saline that contained 2 mg white alum on the 1st day and 7th day, and then they were scheduled to inhale atomized normal saline for 30 min on the 24th day, 25th day and 26th day, and to exposure to filtered air for 3 h on the 27th day; 2) mice in the OVA+air group (asthma group) were treated in much the same way as those in the control group, except that they were injected with 20 μg OVA (grade V, Sigma-Aldrich, USA), rather than white alum, on the 1st day and 7th day, and they and they inhaled atomized 5% OVA, instead of normal saline, on the 24th, 25th and 26th days; 3) mice in the saline+ozone group were also treated in an approach most identically to the control group, except that they were exposed to 2.0 ppm ozone, rather than filtered air, on the 27th day; and 4) mice in the OVA+ozone group were treated in a way much the same as asthma group, except that mice were exposed to 2.0 ppm ozone, rather than filtered air, on the 27th day.
