# Supplementary material for: Exposure to ozone impacted Th1/Th2 imbalance of CD4+ T cells and apoptosis of ASMCs underlying asthmatic progression by activating lncRNA PVT1-miR-15a-5p/miR-29c-3p signaling
Source: Aging (Albany NY). 2020 Nov 20;12(24):25229–55. doi: 10.18632/aging.104124 (PMC7803560; doi:10.18632/aging.104124)
Supplement: Supplementary Tables [file aging-12-104124-s002.pdf]

SUPPLEMENTARY TABLES

Supplementary Table 1. Ozone concentrations in 2016.

Country	Concentrations (ppb)	Country	Concentrations (ppb)
Afghanistan	63	Libya	61
Albania	68	Lithuania	51
Algeria	70	Luxembourg	54
Angola	88	Macedonia	66
Argentina	40	Madagascar	40
Armenia	62	Malawi	53
Australia	36	Malaysia	49
Austria	59	Mali	50
Azerbaijan	59	Mauritania	44
Bangladesh	75	Mexico	59
Belarus	51	Moldova	58
Belgium	54	Mongolia	49
Belize	46	Montenegro	64
Benin	71	Morocco	58
Bhutan	70	Mozambique	48
Bolivia	63	Myanmar	82
Bosnia and Herzegovina	64	Namibia	61
Botswana	59	Nepal	79
Brazil	54	Netherlands	53
Bulgaria	65	New Zealand	35
Burundi	53	Nicaragua	41
Cambodia	51	Niger	52
Cameroon	67	Nigeria	67
Canada	56	North Korea	70
Central African Republic	77	Norway	45
Chad	60	Oman	79
Chile	43	Pakistan	70
China	66	Palestine	72
Colombia	49	Panama	43
Congo	62	Papua New Guinea	29
Costa Rica	41	Paraguay	58
Cote d'lvoire	61	Peru	45
Croatia	65	Philippines	40
Cuba	48	Poland	55
Cyprus	68	Portugal	54
Czech Republic	58	Puerto Rico	42
Democratic Republic of Congo	64	Qatar	117
Denmark	50	Romania	59
Dominican Republic	45	Russia	48
Ecuador	42	Rwanda	52
Egypt	70	Saudi Arabia	69
El Salvador	57	Senegal	45
Equatorial Guinea	53	Serbia	61

Eritrea	47	Sierra Leone	58
Estonia	48	Slovakia	60
Ethiopia	51	Slovenia	64
Fiji	35	Solomon Islands	28
Finland	46	Somalia	40
France	56	South Africa	53
Gabon	51	South Korea	69
Georgia	59	South Sudan	64
Germany	55	Spain	61
Ghana	66	Sudan	51
Greece	69	Suriname	34
Greenland	34	Swaziland	51
Guatemala	57	Sweden	46
Guinea	60	Switzerland	59
Guyana	32	Syria	70
Haiti	47	Taiwan	70
Honduras	45	Tajikistan	64
Hungary	61	Tanzania	47
Iceland	45	Thailand	59
India	77	Timor-Leste	37
Indonesia	44	Togo	72
Iran	74	Tunisia	67
Iraq	75	Turkey	67
Ireland	48	Turkmenistan	59
Israel	71	Uganda	51
Italy	74	Ukraine	57
Jamaica	44	United Arab Emirates	103
Japan	62	United Kingdom	49
Jordan	73	United States	66
Kazakhstan	52	Uruguay	39
Kenya	42	Uzbekistan	63
Kuwait	84	Vanuatu	37
Kyrgyzstan	65	Venezuela	48
Laos	63	Vietnam	56
Latvia	49	Yemen	54
Lebanon	71	Zambia	64
Lesotho	55	Zimbabwe	58
Liberia	55		

Supplementary Table 2. Asthma diagnosis around the world.

Region	Country	Doctor Diagnosed Asthma (%)	Clinical Asthma (%)	Wheezing Symptoms (%)
**Africa**	Burkina Faso	2.02	2.26	5.32
	Chad	3.68	3.94	7.64
	Comoros	7.55	7.8	12.85
	Congo	4.65	4.79	7.93
	Cote d'Iviore	4.22	4.59	7.7
	Ethiopia	2	2	5.53
	Ghana	3.65	3.77	4.88
	Kenya	2.86	3.12	6.22
	Malawi	4.62	4.67	7.76
	Mali	2.65	2.82	4.77
	Mauritania	6.95	7.54	11.78
	Mauritius	3.88	3.92	6.88
	Namibia	3.16	3.39	8.14
	Senegal	3.43	3.72	8.4
	South Africa	5.92	6.09	12.4
	Swaziland	8.74	9.69	15.37
	Zambia	2.83	2.96	6.25
	Zimbabwe	2.28	2.52	5.48
Regional Sub-total		3.94	4.19	7.75
**Americas**	Brazil	12.44	12.98	22.56
	Dominican	9.63	9.97	12.39
	Ecuador	2.03	2.13	3.83
	Guatemala	2.26	2.42	11.95
	Mexico	2.39	2.39	3.87
	Paraguay	6.08	6.4	12.74
	Uruguay	8.6	9.1	12.02
Regional Sub-total		4.27	4.4	7.61
**Eastern Mediterranean**	Morocco	2.76	2.84	11.65
	Pakistan	3.12	3.13	5.02
	Tunisia	2.74	2.79	7.21
	United Arab Emirates	5.3	2.79	7.21
Regional Sub-total		2.93	2.99	7.6
**Europe**	Austria	7.46	7.63	9.48
	Belgium	9.83	10	17.22
	Bosnia Herzegovina	1.3	1.41	4.01
	Crotia	4.38	4.57	8.66
	Czech Republic	4.56	4.71	6.32
	Denmark	9.5	10.19	15.4
	Estonia	2	1.99	6.94
	Finland	9.39	10.24	17.19
	France	10.43	10.59	15.2
	Georgia	2.09	2.15	4.83
	Germany	7.58	7.55	9.25

	Greece	6.6	6.84	10.14
	Hungary	7.66	7.66	14.72
	Ireland	9.41	9.19	11.39
	Israel	7.59	8.54	14.98
	Italy	6.05	6.26	8.98
	Kazakhstan	1.43	1.47	3.36
	Latvia	2.7	2.7	5.9
	Luxembourg	9.16	9.44	16.63
	Netherlands	15.17	15.32	22.71
	Norway	11.05	12.32	15.05
	Portugal	7.83	7.83	8.72
	Russia	2.5	2.57	4.98
	Slovakia	4.11	4.1	7.41
	Slovenia	8.7	8.66	11.91
	Spain	6.79	7.12	12.78
	Sweden	20.09	20.18	21.6
	Turkey	2.06	2.11	11.34
	UK	17.59	18.15	22.59
	Ukraine	2.77	2.9	11.13
Regional Sub-total		5.1	5.28	10.71
**South East Asia**	Bangladesh	2.91	3.23	8.63
	India	3.16	3.3	9.63
	Myanmar	2.36	2.41	3.47
	Nepal	2.04	2.16	14.37
	Sri Lanka	2.6	2.75	6.35
Regional Sub-total		3.24	3.39	9.71
**Western Pacific**	Australia	20.96	21.51	27.39
	China	0.19	1.42	1.73
	Laos	2.72	3.02	5.16
	Malaysia	5.21	5.51	7.55
	Philippines	7.21	7.46	11.01
	Vietnam	0.82	1.04	2.05
Regional Sub-total		5.85	6.17	8.88

Supplementary Table 3. Comparison of baseline characteristics among asthma patients of acute stage, asthma patients of remission stage and healthy controls.

Clinical characteristics	Asthma acute group (N=85)	Asthma remission group (N=62)	Healthy control group (N=46)
Age (year)	38.76±10.52	40.42±8.37	41.25±9.06
Gender			
Female	40	32	21
Male	45	30	25
Disease Course (year)	5.62±3.28	6.03±4.15	-
FEV1(%)	55.26±7.68*#	81.52±9.18*	90.91±6.99
FEV1/FVC(%)	64.50±8.15*#	75.55±6.71*	86.16±10.11
TNF-α (ng/L)	2.81±0.29*#	1.41±0.27*	0.33±0.16
IL-13 (ng/L)	35.68±6.82*#	24.01±4.38*	8.42±3.06
IL-4 (ng/L)	115.37±38.26*#	55.24±17.54*	41.93±13.16
IL-10 (ng/L)	96.43±19.57*#	49.31±13.08*	36.73±9.31
IFN-γ (ng/L)	33.89±6.87*#	54.62±8.42*	69.31±9.59
IL-2 (ng/L)	118.64±38.14*#	186.32±43.13*	207.45±50.12
hs-CRP (mg/L)	13.18±3.25*#	5.22±1.03*	1.47±0.40
FeNO (ppb)	92.47±28.14*#	42.39±17.12*	14.84±4.25

* $p<0.05$ when compared with healthy control group; # $p<0.05$ when compared with asthma remission group.

Supplementary Table 4. Diagnostic efficiency of lncRNA PVT1 for asthma.

Group	Value	Sensitivity	Specificity	AUC	95% CI
Asthma vs. Group	2.83	0.844	0.978	0.909	0.87-0.95
Acute vs. Remission	7.32	0.518	0.855	0.705	0.62-0.79

Supplementary Table 5. The sequences of the primers for the amplification used by real-time PCR.

microRNA	Primers (5'-3')	
	Forward	Reverse
hsa-miR-15a-5p	ATCCAGTGCGTGTCGTG	TGCTTAGCAGCACATAATG
hsa-miR-140-5p	CCCCCAGTGGTTTTACCCTA	GTGCGTGTCGTGGAGTCG
hsa-miR-20b-5p	TGTCAACGATACGCTACGA	GCTCATAGTGCAGGTAGA
hsa-miR-488-3p	CGGGGCAGCUCAGUACAG	CAGTGCGTGTCGTGGAGT
has-miR-455-5p	CGAGCTTCCTTCTGCAGGT	CACCACTGCCATCCCACA
hsa-miR-29c-3p	GCCTAGCACCATTTGAAATCG	GTGCAGGGTCCGAGGT
hsa-miR-143-3p	GGGGTGAGATGAAGCACTG	CAGTGCGTGTCGTGGAGT
hsa-miR-511-3p	GTCTTTTGCTCTGCAGTC	GAACATGTCTGCGTATCTC
hsa-miR-497-5p	CCTTCAGCAGCACACTGTGG	CAGTGCAGGGTCCGAGGTAT
